# Neurologic manifestations of COVID-19: what can we learn from other coronaviruses

**DOI:** 10.1186/s41983-020-00240-w

**Published:** 2020-11-11

**Authors:** Zohreh Jadali

**Affiliations:** Department of Immunology, School of Public Health, Tehran University of Medical Sciences, P.O.BOX: 6446, Tehran, 14155 Iran

**Keywords:** Pathogenesis, Coronavirus, SARS-CoV-2, Neurological complications, Cytokines

To the Editor,

The current pandemic that has paralyzed the world economy is caused by a new coronavirus [severe acute respiratory syndrome coronavirus 2 (SARS-CoV-2)]. Although the lung is a major target organ of the virus, it can also strike other parts of the body including the nervous system. Neurologic presentations of COVID-19 are ranging from nonspecific and moderate to severe. Most patients have mild symptoms like headache and make a good recovery. However, a minority of patients develop severe complications with unfavorable outcomes including cerebrovascular diseases [1].

SARS-CoV-2 is considered novel because of its special features. But simultaneously, there are similarities between this and other betacoronaviruses. For instance, genomic analysis indicated that Middle East respiratory syndrome coronavirus and SARS-CoV share high degrees of sequence homology with SARS-CoV-2. Moreover, SARS-CoV and SARS-CoV-2 use the same host receptor for cell entry [2]. Like other coronaviruses, SARS-CoV-2 is neurotropic and may spread to the nervous system via similar mechanisms.

Neurotropic and neuroinvasive properties of SARS-CoV-2 are supported by several observations including the presence of virus particles in the cerebrospinal fluid of patients with significant nervous system symptoms [3].

Currently, it is difficult to distinguish whether neurological complications of COVID-19 are a consequence of direct or indirect effects of viral infection. However, existing data highlight the importance of considering both effects.

Direct effects may occur by multiple mechanisms including viral transit across the cribriform plate or olfactory bulb to brain. Another mechanism relies on blood circulation and angiotensin-converting enzyme 2 (ACE2) receptors that are expressed on glial cells, neurons, and capillary endothelium and are involved in virus entry [4].

In addition to direct adverse consequences, several indirect mechanisms have been proposed to account for neuropathologic properties of SARS-CoV-2. For instance, viral replication within the lung pneumocytes may lead to hypoxic conditions and gas exchange disorders in the nervous system. Another mechanism appears to be related to ACE2 which acts as a cardiocerebral vascular protection factor and plays an important role in blood pressure regulation and antiatherosclerosis mechanisms. Virus binding to ACE2 receptors can lead to hypertension and increase the risk of ischemic stroke and cerebral hemorrhage [1, 4].

The third indirect mechanism is linked to the host immune response against virus. Some evidence indicates that neurological symptoms of COVID-19 could be induced by uncontrolled production of cytokines. These mediators are produced by different cells including glial cells.

The ability of these cells to produce proinflammatory cytokines has also been demonstrated in experimental models, after corona virus infection [4]. Molecular mimicry that could be associated with the development of autoimmunity is another mechanism by which SARS-CoV-2 may trigger an immune response against nervous system-specific proteins [5]. These phenomena may explain why some COVID-19 patients can develop disabling neurologic disorders including Guillain-Barré syndrome.

Altogether, the nervous system may be affected by SARS-CoV-2. Neurological manifestations related to the virus are varied and complex. Therefore, understanding of neural pathways that contribute to neurologic deficits can be beneficial in promoting assessment and management of COVID-19 patients.

## Data Availability

Data is available whenever requested.

## Abbreviations

SARS-CoV-2: Severe acute respiratory syndrome coronavirus 2
ACE2: Angiotensin-converting enzyme 2
